# Case report: Subjective loss of performance after pulmonary embolism in an athlete– beyond normal values

**DOI:** 10.1186/s12890-016-0180-x

**Published:** 2016-01-28

**Authors:** Daniel Dumitrescu, Felix Gerhardt, Thomas Viethen, Matthias Schmidt, Eckhard Mayer, Stephan Rosenkranz

**Affiliations:** Klinik III für Innere Medizin, Herzzentrum der Universität zu Köln, Kerpener Str. 62, 50937 Köln, Germany; Klinik für Nuklearmedizin, Universitätsklinikum Köln, Cologne, Germany; Department of Thoracic Surgery, Kerckhoff-Clinic, Bad Nauheim, Germany

**Keywords:** Chronic thromboembolic disease, Pulmonary hypertension, Pulmonary endarterectomy, Pulmonary embolism, Cardiopulmonary exercise testing

## Abstract

**Background:**

Chronic thromboembolic pulmonary hypertension (CTEPH) is a progressive disease. For patients with operable CTEPH, there is a clear recommendation for surgical removal of persistent thrombi by pulmonary endarterectomy (PEA). However, without the presence of PH, therapeutic management of chronic thromboembolic disease (CTED) is challenging - especially in highly trained subjects exceeding predicted values of maximal exercise capacity.

**Case presentation:**

A 43-year-old male athlete reported with progressive exercise limitation since 8 months. Six months earlier, pulmonary embolism had occurred, and was treated since with oral anticoagulation. A pulmonary ventilation/perfusion scan showed severe ventilation/perfusion mismatch: chest CT and pulmonary angiography revealed bilateral wall-adherent thrombotic material, but pulmonary hemodynamics were completely normal. His peak oxygen uptake exceeded predicted values, however exercise ventilatory efficiency was abnormal, compared to a matching athlete. After thoroughly discussing therapeutic options with the patient, he successfully underwent pulmonary endarterectomy at an expert center. Five and twelve months after surgery, his maximal exercise capacity and ventilatory efficiency profoundly improved beyond preoperative values, and his subjective exercise tolerance had returned to normal.

**Conclusions:**

Significant CTED may be present without relevant pathologic changes in pulmonary hemodynamics at rest. Reaching normal values of maximal exercise capacity does not exclude pulmonary vascular disease in highly trained subjects. More data are needed to evaluate the risk-/benefit ratio of PEA in patients with CTED and normal pulmonary hemodynamics. A thorough discussion with the patient as well as shared decision making regarding therapy are mandatory. Cardiopulmonary exercise testing may add important clinical information in the non-invasive diagnostic evaluation at baseline and during follow-up.

## Background

Chronic thromboembolic pulmonary hypertension (CTEPH) is a progressive disease caused by wall-adherent, fibrotic thromboembolic material in the precapillary pulmonary circulation, despite oral anticoagulation. For patients with operable CTEPH, there is a clear recommendation for surgical removal of persistent thrombi by pulmonary endarterectomy (PEA) as a curative approach in specialized centers [1, 2]. However, without the presence of PH, therapeutic management of chronic thromboembolic disease (CTED) is challenging [3]. Currently, there is only rare evidence on therapeutic strategies of CTED and normal hemodynamics. In selected cases such as in athletes, reduced exercise tolerance and dyspnea may be related to the extent of persistent pulmonary perfusion defects and dead space ventilation, despite exceeding predicted values of maximal exercise capacity and normal pulmonary hemodynamics.

## Case Presentation

We report the case of a 43-year-old male athlete (190 cm, 82 kg) who presented with progressive dyspnea and subjective exercise limitation in the prior 8 months. He was able to cycle a distance of 50 kilometers per day and reported a continuously decreased ability to sustain this effort. Other daily activities were not affected. He had suffered from pulmonary embolism 6 months prior to presentation, and had permanently been on therapeutic oral anticoagulation since this time point. He did not report any other known diseases.

Physical examination did not reveal any relevant findings. Pulmonary function testing did not show any restrictive or obstructive limitation, with normal diffusion capacity. Echocardiography showed normal values, except a slightly enlarged right ventricle (basal diameter 42 mm) and a slightly enlarged right atrium (area 19.8 cm^2^). No tricuspid regurgitation could be detected.

**Fig. 1:**
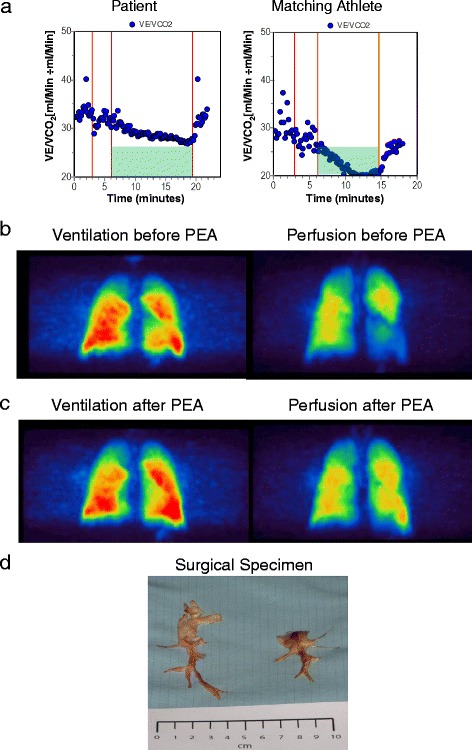
Functional assessment and imaging of chronic thromboembolic material in the pulmonary circulation. **a** VE/VCO_2_ ratio during exercise. Lower values indicate better ventilatory efficiency. Compared to a matching athlete (male, 46 y, 177 cm, 70 kg), impaired ventilatory efficiency is visible, reflecting ventilation/perfusion mismatch. **b** Ventilation-/perfusion (V/Q) scan showing large defects in the right upper lobe and in the left lower lobe. **c** V/Q scan 12 months after PEA, showing a substantial reduction of previously documented perfusion defects. **d** Surgical specimen. Successful bilateral pulmonary endarterectomy of wall-adherent thrombotic material with advanced fibrotic remodelling

The patient’s maximal exercise performance during cardiopulmonary exercise testing (CPET) was excellent, compared to his predicted values. He exercised on a cycle ergometer with a ramp protocol of 25 Watts per minute, and achieved 115 % of his predicted peak oxygen uptake. Ventilatory efficiency was normal, relative to predicted values for an untrained, adult population [4]. However, compared to an athlete with a matching exercise capacity, his ventilatory efficiency during exercise was reduced, expressed by an elevated ratio of minute ventilation to CO_2_ output (VE/VCO_2_), indicating a ventilation-/perfusion mismatch during exercise (Fig. 1a). A pulmonary ventilation/perfusion scan revealed large bilateral perfusion defects (Fig. 1b), which could be confirmed by CT scan. Surprisingly, the patient’s pulmonary hemodynamics were completely normal. Mean pulmonary arterial pressure was 18 mmHg, with a pulmonary arterial occlusion (wedge) pressure of 7 mmHg, a cardiac index of 3.85 L/Min/m^2^ and a pulmonary vascular resistance of 1.35 wood units. Additionally, invasive exercise hemodynamics were obtained at a workload of 200 Watts. Mean pulmonary arterial pressure increased up to 43 mmHg, however with a constant pulmonary arterial occlusion pressure and a substantial increase in cardiac output, so that pulmonary vascular resistance remained slightly unchanged at 1.56 wood units. Current guidelines do not recommend the use of the term ‘exercise induced PH’ [1], as there is not sufficient evidence to support the definition. A current publication suggests to define a pathological exercise hemodynamic response as an increase of mean PAP above 30 mmHg, combined with a cardiac output of less than 10 L/Min, or with a PVR of less than 3 Wood units (240 dyn x sec x cm^-5^) [5]. However, according to this definition, even exercise hemodynamics were normal in this case.

For this situation, currently no evidence-based therapeutic recommendations are available. One single-center study retrospectively analyzed all patients with CTED and normal hemodynamics who had undergone surgery [3]. Almost all patients improved in functional class, however changes in six-minute walking distance (6MWD), were heterogeneous. Although none of the patients died, major complications occurred in 40 % of the cases.

In the present case, therapeutic options were thoroughly discussed with the patient. He expressed a high degree of suffering. Considering the progressive nature of the disease, and in consensus with the patient’s request, he was referred to an experienced center for PEA. Pulmonary angiography confirmed surgical accessibility of the thromboembolic material. The patient successfully underwent surgery 3 months after initial presentation (surgical specimen, Fig. 1d). Lifelong oral anticoagulation was recommended. An inferior vena cava filter (IVC) was not inserted. There were no complications during the PEA procedure or during the post-operative phase. The patient was discharged from hospital 10 days after the procedure.

**Fig. 2:**
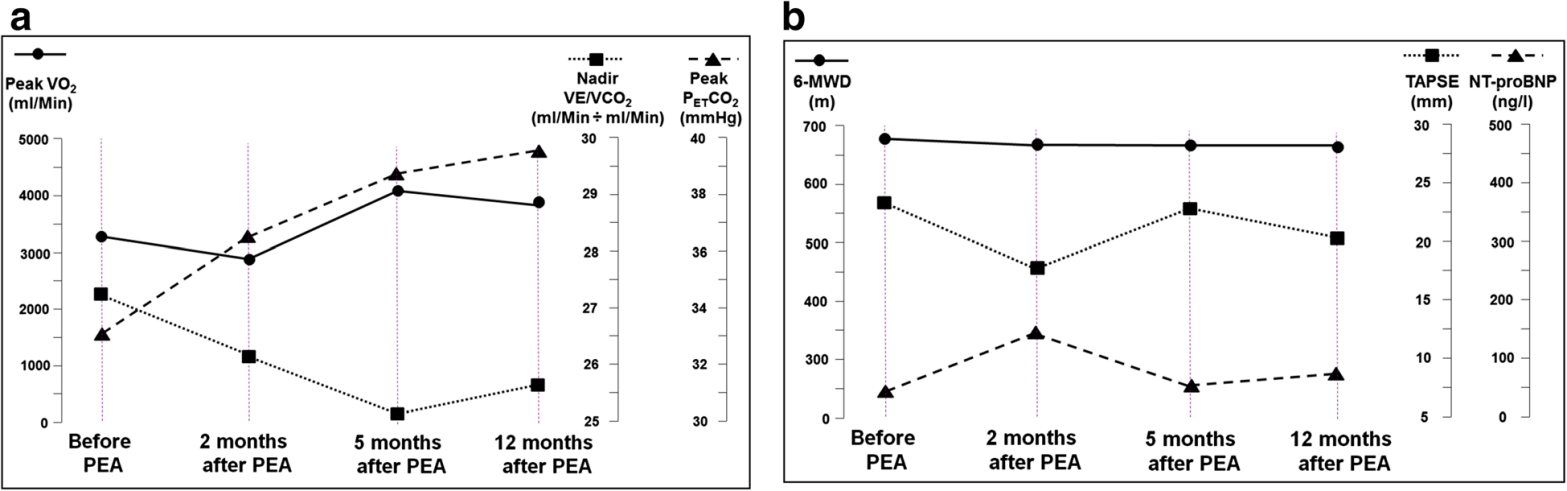
Non-invasive serial measurements in a 43-year old, athletic patient with CTED and normal pulmonary hemodynamics. **a** CPET results. Constant improvement of ventilatory efficiency after surgery, reflected by decreasing VE/VCO_2_ ratio and increasing peak P_ET_CO_2_ values. Delayed improvement of peak VO_2_. **b** Results of other non-invasive diagnostic tools established for serial follow-up in PH. Delayed improvement of ventricular function. Constant results of 6-minute walking distance

Two months after PEA, the patient was re-evaluated by echocardiography and CPET. At this time point, the patient did not feel any subjective improvement. Right ventricular dimensions were unchanged, however tricuspid annular plane systolic excursion (TAPSE) was reduced to 16 mm – compared to 22 mm before PEA. Peak oxygen uptake was reduced to 94 % of predicted values, however ventilatory efficiency had improved, reflected by a lower VE/VCO_2_ ratio (Fig. 2a).

However, five months after PEA, a significant treatment success could be documented. The patient reported to feel better, and peak oxygen uptake significantly improved to 4.0 L/Min (136 % of predicted values). Ventilatory efficiency was not normalized but further improved, documented by a lower VE/VCO_2_ ratio compared to all previous tests (Fig. 2a).

One year after PEA; a follow-up ventilation-/perfusion scan showed minor residual defects (Fig. 1c). In accordance with these findings, CPET showed a sustained improvement of peak oxygen uptake and of ventilatory efficiency, reflected by a decreased VE/VCO_2_ ratio and substantially higher peak P_ET_CO_2_ values during exercise (Fig. 2a). TAPSE and NTpro-BNP levels had returned to normal (Fig. 2b).

Follow-up measurements in patients with CTED and normal pulmonary hemodynamics are challenging. Currently, there is only one published case report in the literature [6]. Here, therapeutic effects were documented by CPET during serial follow-up visits. A substantial improvement in maximal exercise performance and in ventilatory efficiency could be demonstrated six months after surgery.

At present, there are no data showing *early* effects after PEA in patients with normal hemodynamics. However, insights gained from CTEPH suggest that restoration of right and left ventricular function may be delayed [7, 8].

## Conclusion

This case remarkably demonstrates that significant chronic thromboembolic disease may be present without relevant pathologic changes in pulmonary hemodynamics at rest. Cardiopulmonary limitation may be masked by a high level of physical fitness. Thus, reaching normal values of maximal exercise capacity does not exclude pulmonary vascular disease in highly trained subjects. CPET may add clinical information on the degree of pulmonary ventilation-/perfusion mismatching during exercise.

Although pathophysiological considerations suggest a beneficial treatment effect, more data are needed to evaluate the risk-/benefit ratio of PEA in patients with CTED and normal pulmonary hemodynamics. A thorough discussion with the patient as well as shared decision making regarding therapy are mandatory. Patients should only be referred to PEA expert centers. Successful surgical removal of chronic thromboembolic material may lead to beneficial, sustained treatment effects even in patients with normal pulmonary hemodynamics. However, the onset of these effects may be delayed. CPET may adequately reflect the response to therapy over time.

### Consent

Written informed consent was obtained from the patient for publication of this case report and any accompanying images.

## Abbreviations

6MWD: 6-minute walking distance
CO_2_: Carbon dioxide
CPET: Cardiopulmonary exercise testing
CT: Computed tomography
CTED: Chronic thromboembolic disease
CTEPH: Chronic thromboembolic pulmonary hypertension
NT-pro BNP: N-terminal fragment of brain natriuretic peptide
PEA: Pulmonary endarterectomy
P_ET_CO_2_: End-tidal carbon dioxide partial pressure
TAPSE: Tricuspid annular plane systolic excursion
V/Q: Ventilation/perfusion
VE: Minute ventilation
VCO_2_: Carbon dioxide output
VE/VCO_2_: Ratio of minute ventilation to carbon dioxide output
